# Use of non-intrusive laser exfoliation to improve substance uptake into citrus leaves

**DOI:** 10.12688/f1000research.129789.3

**Published:** 2023-05-10

**Authors:** Luis Ponce Cabrera, Ed Etxeberria, Pedro Gonzalez, Teresa Flores Reyes

**Affiliations:** 1 Onteko LLC, Olive Branch, MS, 38654, USA; 2 IFAS, Citrus Research and Education Center, University of Florida, Lake Alfred, FL, 33850, USA

**Keywords:** Citrus, foliar sprays, foliar uptake, laser light

## Abstract

**Background: **Despite the presence of stomata in leaves, foliar application of agrochemicals can be extremely inefficient due to the low permeability of leaf cuticular surfaces to polar compounds.

**Methods: **This study introduced a laser-based “wax exfoliation” method to facilitate the penetration of substances into the leaf and, together with enhancing their uptake into the phloem and subsequent transport across tissue. This investigation demonstrated the effectiveness and non-invasive properties of laser exfoliation to improve the penetration of foliar-applied substances into citrus leaves.

**Results: **This work presents the use of laser energy to exfoliate the cuticle of a leaf, with the highest energy density of 0.76 J/ cm2 resulting in 85-90% exfoliation across the entire laser-spot area. The infrared wavelength of the erbium laser is specifically chosen to target the wax cuticle without causing damage to the underlying epidermal cells. This selective ablation allows for increased penetration of therapeutic compounds into the leaf and transportation throughout the plant's vasculature. This is demonstrated using a fluorescent glucose analog applied to the laser treated leaves, showing increased penetration and transport throughout the leaf.

**Conclusions: **Our findings demonstrate that the use of laser technology for the foliar application of agrochemicals provides significant advantages, including improved foliage uptake of therapeutic compounds. The method of cuticle exfoliation presented in this study is highly effective and non-intrusive, limiting its effects to the cuticle only. Future work should focus on the development of prototypes for in-field applications, including testing at longer distances as the Er:YAG laser does not require a lens for this application.

## Introduction

One of the most prevalent methods used in modern-day agriculture to improve crop health, and hence yield, is the foliar application of agrochemicals. ^1^ However, several barriers cause retardation and interfere with the efficient penetration and utilization of these substances. For example, the leaf surface is coated by a waxy cuticle that serves as a barrier for the prevention of water loss and pathogenic entry into the plant body. Due to its water impermeable nature, the cuticle also prevents the entry of externally applied soluble compounds such as most agrochemicals. The movement of substances into the leaf occurs primarily through the stomata, ^2^ located mainly on the less-exposed abaxial side of plant leaves. ^3^ This property reduces the functional surface area leading to reduced agrochemical penetration through the foliar route. As consequence, considerable quantities of applied chemicals end up in the plant’s natural environment and can generate undesirable ecological impacts.

An example of research focused on foliar application of substances into plant leaves is the study of agrochemicals to treat citrus Huanglongbing (HLB), ^4^^,^^5^ a bacterial disease of citrus that has spiraled the Florida citrus industry to the brink of disappearance and actively spreading across other regions of the world on a large scale. ^6^ In the case of HLB infections, therapies considered against the causative bacterium *Candidatus liberibacter* (CLas) ^7^ include treatment with antimicrobial agents as a promising alternative to enhance the crop’s lifespan. ^5^ However, response to such treatments is diminished due to challenges associated with the application process and the ensuing slow penetration into the phloem, the conductive tissue where bacterial populations aggregate within infected citrus plants. ^8^

A novel alternative was recently identified to enhance penetration of agrochemicals into the leaf. This system involves the generation of leaf perforations of approximately 250 μm in diameter using a CO _2_ laser directly focused upon the leaf surface. The perforations not only puncture the cuticle but also perforate the epidermis and few layers of underlying palisade parenchyma. ^7^ When applied on treated leaves, penetration of sample substances was increased over 2,000% over untreated leaves. ^9^

The use of laser micro-perforations as a plant pharmacodynamics-enhancing technique has several drawbacks. First, apart from the cuticle, laser-induced pores also affect the underlying leaf epidermis and palisade parenchyma due to the inevitable removal of material from both areas. ^9^^,^^10^ Second, this methodology requires highly focalized lasers to achieve maximum efficiency within field conditions, which is technologically complex due to the intrinsic irregularity of leaf surfaces, their random orientation, and depths within a tree.

The goal of this investigation was to devise a lesser invasive mechanism to enhance substance uptake into the leaves using specific laser beams. Our study describes a novel methodology for enhancing the penetration of agrochemicals into citrus leaves without the drastic requirement for physically perforating leaf tissues. This novel technique is based upon epidermal water content-dependent selective absorption of Erbium laser light. Partial separation of the waxy cuticle is successfully obtained across an area of several square centimeters through the application of a single laser shot. Since there is no damage afflicted to the leaf epidermal tissue, the cuticle rapidly regenerates within a brief period, thereby recovering its protective functions.

## Methods

### Plant material

‘Valencia’ orange leaves from greenhouse-reared plants of approximately one meter were selected. ‘Valencia’ orange is one of the main species affected by Huanglongbing (HLB) disease, and its leaves are covered by a thick cuticle layer that can impede the absorption of foliarly applied treatments, making it an ideal model organism. Laser treatments were applied to attached leaves and these remained on the tree for post-laser application in order to provide the live conditions. Treated leaves were consequently detached and transported to the laboratory for further analysis.

### Energy levels

It is well-established that leaves have a high-water content including the epidermis. Since water has a strong absorption band in the 3000 nm wavelength region, an in-house Er:YAG laser (fundamental wavelength = 2940 nm; 200 μs pulse duration) was used for foliage irradiation from a distance of approximately 30 cm. Each laser treatment consisted of a single shot performed without specific focusing of the laser on the leaf surface. In order to discern the varying effect of laser intensity on the leaf wax cuticle three laser energy levels were employed, with a spot area of 0.78 cm ^2^. This spot area was determined by the diameter of the laser crystal used in the experiment (1cm), as no focusing element was employed. This allowed for a direct measurement of the effect of laser intensity on the leaf wax cuticle, without the added variable of a focusing element. No lens was used to focalize the laser and the beam divergence was 0.1 mrad, consequently disregarding the necessity for standardizing irradiation distance in such assays. In all cases, one single pulse sufficed to obtain the effect of wax cuticle exfoliation. The laser energies tested for the removal of wax across the investigated leaf foliage ranged from 0.3-0.76 J/cm ^2^, depending upon the laser energy level.

### Penetration of applied soluble fluorescent marker

To visualize the penetration of applied substances into the leaf, a fluorescent analog of glucose solution (NBDG: 2-NBDG = 2-[N-(7-nitrobenz-2-oxa-1,3-diazol-4-yl)amino]-2-deoxyglucose. λ Ex/Em (nm) 465/540) was applied to the leaf surface. The NBDG solution was prepared and used at a concentration of 30 mM. ^9^

Microscopic observations were performed using a Carl Zeiss™ Axio Scope A-1 ^®^ microscopy platform equipped with a Canon™ EOS Rebel T3i ^®^ camera and a Carl Zeiss™ AxioCam ICc 1 ^®^. Low magnification images were obtained using a Zeiss™ Stemi SV11 ^®^ fluorescent stereoscope (Carl Zeiss Microscopy™+ GmbH, Göttingen, Germany).

### Imaging

For High-magnification observations of the plant samples with fluorescence, a Carl Zeiss AxioScope A1 fluorescent microscope (Carl Zeiss Microscopy GmbH, Göttingen, Germany) equipped with a Zeiss Axio Cam ICc1, with filter Set 43 or Rhodamine filter from Zeiss (Ex: BP 545/25, Em: BP 525/50) for red and green fluorescence, was used.

Low magnification images were observed under a Wild Heerbrugg stereoscope (Wild Heerbrugg Instruments, Ltd., Heerbrugg, Switzerland) using a Green –Only bandpass filter, Stereo Microscope Fluorescence Adapter SFA-LFS-GO (NIGHTSEA, Electron Microscopy Sciences, Hatfield, PA. 19440). Images were captured with a Canon PowerShot S3 IS (Martin Microscope Co., Easley, SC). The procedures and techniques described in this manuscript have been thoroughly described to allow for replication of the results. All relevant materials, steps, and parameters have been included to ensure full reproducibility of the experiment.

## Results

**Figure 1.**
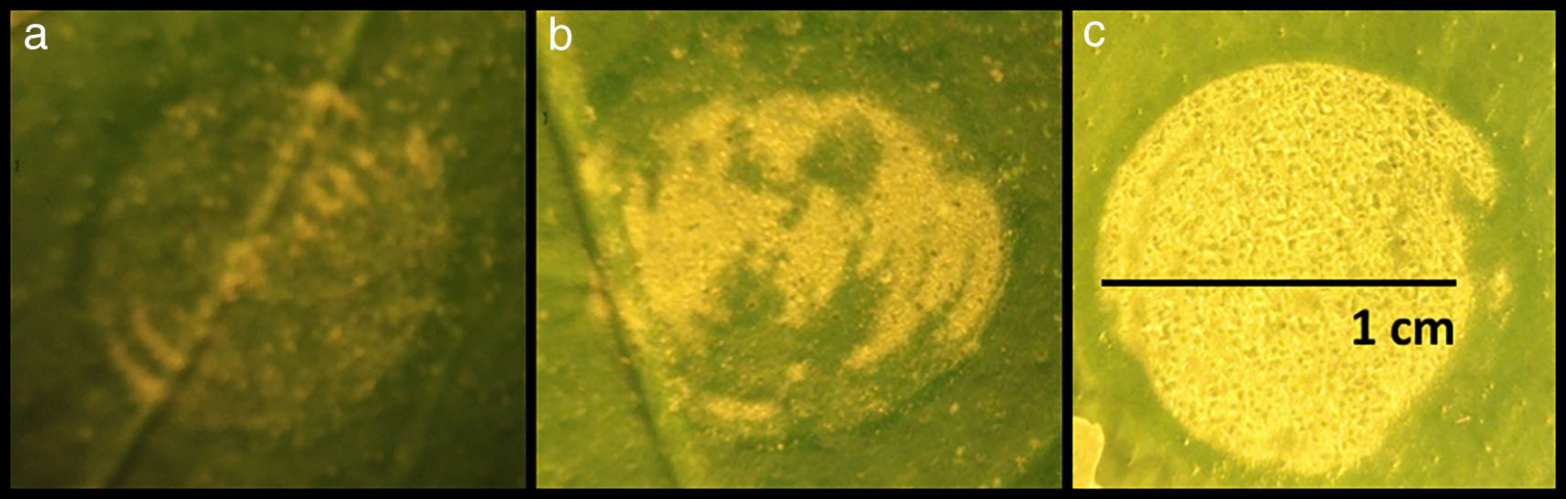
Effect of laser irradiation on citrus leaf surfaces. Figure depicts three different levels of laser energy-intensity. a) 0.3 J/cm ^2^, b) 0.5 J/cm ^2^, c) 0.76 J/cm ^2^.

The effect of increasing laser energy levels applied to the leaf surface was studied by treating 20 leaves from the same tree with three different energy levels. The results of these pulses on three representative leaves are presented in Figure 1. The yellow areas in Figure 1 represent the modified leaf cuticle according to the level of energy applied. The yellow color displayed in the irradiated area is due to the separation of the wax cuticle from the epidermis in that region, altering the reflection of the microscope’s white light. In contrast, for the non-irradiated areas, the transparent wax cuticle remains in contact with the epidermis, which is green in color due to the chlorophyll. Exfoliation of the leaf cuticle increased proportionally with laser intensity level. For the highest energy density value at 0.76 J/cm ^2^, the exfoliation effect was achieved across (85-90%) the entire laser-spot area ( Figure 1c).

**Figure 2.**
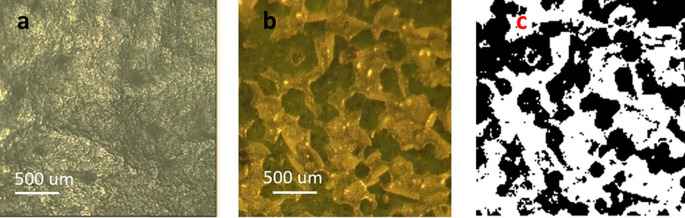
a) Leaf image prior to laser treatment. b). Leaf image post-laser treatment. The green areas are the leaf epidermis seeing through openings created by the laser exfoliation process. c) Black-and-white image post-processing of color images using Power Point™ ‘color’ tool.

A close-up of a treated portion of a leaf using 0.76 J/cm ^2^ energy density is shown in Figure 2. The waxy material, which appears relatively smooth in untreated leaves ( Figure 2a), aggregated and segregated after treatment, forming clean breaches into the epidermis ( Figure 2b). Under these conditions, only the cuticle was affected as the green underlying epidermal layer of cells remained undisturbed by the energy applied. The image in Figure 2c (black-and-white) was generated using the color tool from Power Point ^®^ (Microsoft™, USA) to facilitate the estimation of percent exposed areas. Regarding such estimations, the online tool ‘Coolphptools’ was employed, ^11^ to calculate the percentage area per color. The black-color areas, which corresponded to wax cuticle exfoliated regions, was estimated to cover as 0.61, indicating a 61% success rate in cuticle exfoliation of the total laser-irradiated area. Table 1 shows the average percentage of exfoliated area for each energy level.

**Table 1.** Average exfoliated area for each energy level. It can be appreciated that the exfoliated are is correlated to the energy density of the pulse.

Energy density (J/cm ^2^)	% Exfoliated area	
	Mean	Std Dev
0.3	18.3	7
0.5	37.3	6.1
0.76	61	4.5

The infrared emission wavelength of 2940 nm from the erbium laser coincides with the maximum of the absorption peak of water. Therefore, given the high-water content of the leaf, the absorption of the laser energy is very high.

This phenomenon provokes the rapid heating and exerting of pressure that ‘pushes’ the wax cuticle outwards. Since such a laser-energy/wavelength is not absorbed within the epidermal tissue or any other plant constituent, consequently, there is no irreversible physical damage imposed upon the leaf structural integrity, apart from cuticle exfoliation (temporary separation). The mechanism of selective ablation of plant parts/elements was previously utilized for the removal of cactus spines, where it is possible to pulverize and extract such spines through rapid heating of water content present within glochids. ^12^

**Figure 3.**
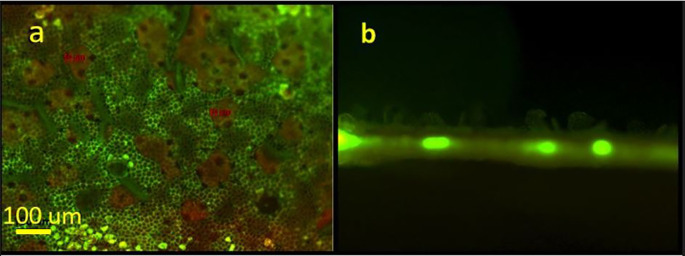
Fluorescent microscopy images of the laser-based wax cuticle exfoliation from a citrus leaf: (a) the treated area of the abaxial of the leaf (upper side) is in green. The yellow -green points at the bottom of the figure correspond to the underlying epidermal walls autofluorescence; (b) Cross-section of a leaf’s laser treated area. Fluoresce of the Vascular bundles are visible.

Figure 3 presents a fluorescent view of a laser treated leaf using a green filter. In Figure 3a, the areas where the cuticular wax was “lifted” or exfoliated appear green in color. The fluorescent green color represents autofluorescence of undamaged epidermal cell walls. These open irregular-shaped areas range from several tens to hundreds of micrometers. The beige areas correspond to the exfoliated wax clumps. A cross section of a treated area in presented in Figure 3b. The figure distinctly shows the “lifting” of the epidermal wax on the upper side of the leaf. The bright spots represent autofluorescence of the vascular tissue.

**Figure 4.**
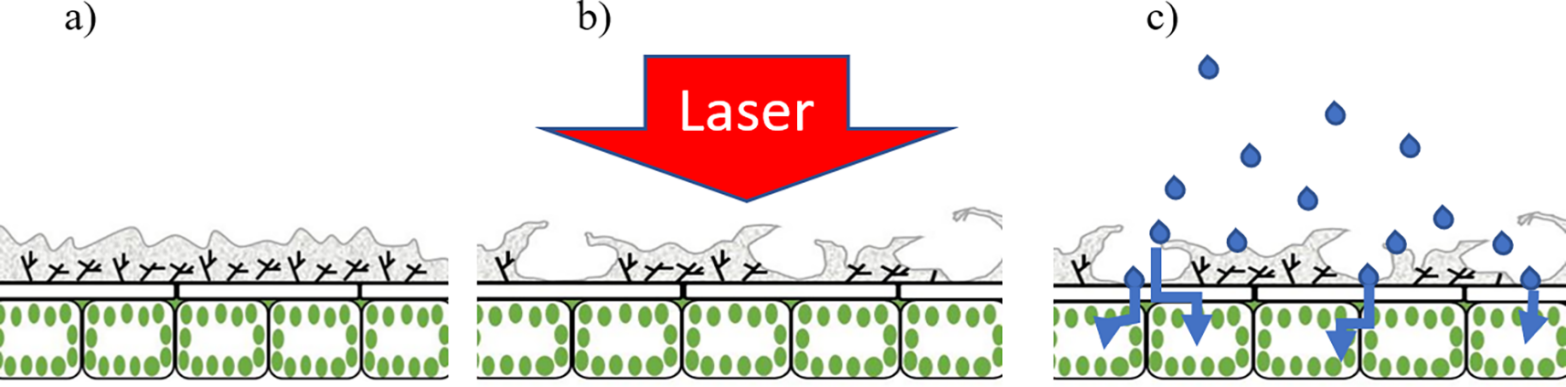
Graphical representation of the laser-based wax cuticle exfoliation methodology; (a) prior to laser ablation, (b) Immediate effect of laser impacting the leaf surface and generating accessibility for therapeutic compounds through wax cuticle exfoliation, (c) Therapeutic compound application and penetration through cuticle-exfoliated areas, successfully reaching the epidermal layer.

Figure 4 is a diagrammatic representation of a laser treated leaf highlighting the leaf epidermis prior to ( Figure 4a) and following ( Figure 4b) application of a laser pulse. In Figure 4, the grey areas represent the cuticle whereas the underlying cells include the epidermal cells and the palisade cells. Post-laser treatment, the cuticle appears ‘lifted’ (or partially detached) as a result of the pressure exerted by the laser-excited water ( Figure 4b). Through such areas having detached and raised cuticles, therapeutic compounds can easily penetrate into the leaf epidermal layer and then follow its pathway to the plant transport system.

**Figure 5.**
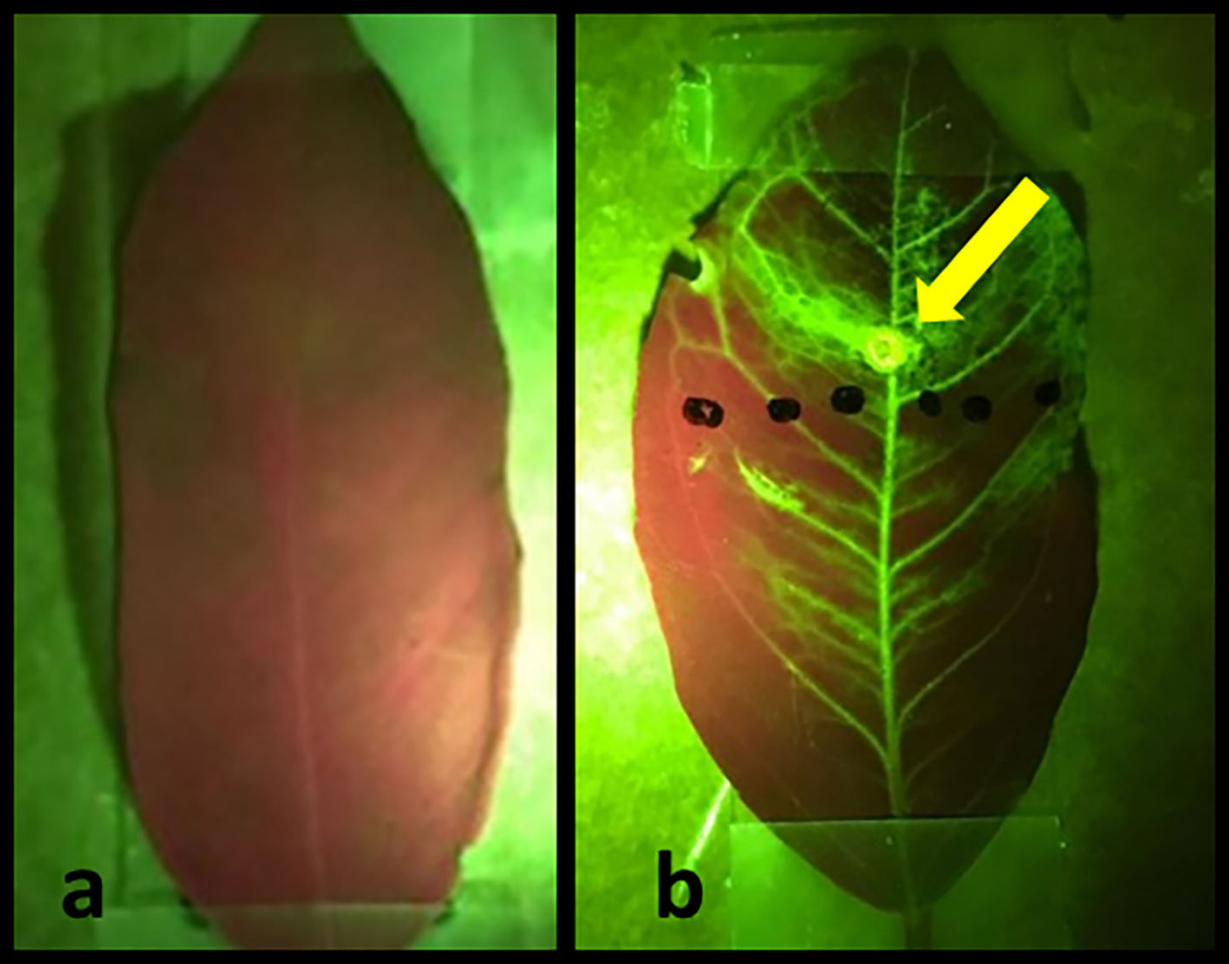
Images of citrus leaves green/red. (a) control untreated leaf, and (b) laser-treated leaf two hours after application of fluorescent NBDG. The green spot indicated by the arrow is the laser spot application area. Figures were taken using an I-phone with a green-fluorescent filter.

To demonstrate the effectiveness of the laser treatments in allowing externally applied hydrophobic substances to penetrate the leaf and travel throughout the plant vasculature, we applied a fluorescent NBDG to laser treated areas ( Figure 5). The images are viewed under green/red control untreated leaf is presented in Figure 5a. Conversely, Figure 5b depicts a treated leaf two hours after laser treatment and NBDG application. The externally applied fluorescent NBDG is visible throughout the leaf, especially in the veins containing the plant vascular tissue, clearly indicating successful solution penetration and distribution across the majority of leaf surface area.

## Conclusion

The application of agrochemicals through the foliar route remains a “gold-standard” therapeutic administration route for enhancing crop productivity, treatment of diseases, and pathogen/parasite circumvention and prophylaxis. However, despite its wide application, penetration through leaves remains quite inefficient, causing dramatical environmental impact as > 90% of applied agrochemical doses by the foliar route are not absorbed by the plant and eventually lead to a detrimental impact on the immediate plant environment. ^1^ The use of laser technology for foliar application of agrochemicals contributes with a plethora of advantages. Aside from the improving foliage uptake of most therapeutic compounds/agrochemicals, it results in the reduction in agrochemical losses/wastage and eventual detrimental impact on the treated plant’s immediate environment. The high efficacy provided by the cuticle exfoliation method presented in this communication eliminates perforation of live tissue by limiting its effect to the cuticle only, therefore, providing a less intrusive method for substance penetration. Future work requires the development of field application prototypes capable of operating at longer distances. We conducted experiments using an extremely simple optical arrangement that did not require a lens to focus the laser. Although the simplicity of the optics for long distances still needs to be verified, an estimate can be made based on simple calculations. For an Erbium laser with M2 < 2, we would have an angular divergence of 1.49 mrad. This means that for a distance of 5 meters from the aperture to the treatment area, we would have a beam diameter of approximately 1.48 cm and a spot area of 1.72 cm ^2^. For this area, an energy density of 0.7 J/cm ^2^ could be achieved with pulses of 1.2 J of energy. A treatment distance of 5 meters would be sufficient for use under field conditions, in combination with substance sprayers currently used in the industry.

## Data Availability

Open Science Framework: Use of non-intrusive laser exfoliation to improve substance uptake into citrus leaves. DOI: 10.17605/OSF.IO/6M43P. ^13^ This project contains the following underlying data:
• Statistics exfoliated area (Percentage exfoliated area and pulse energy density for all samples in this study.) Data are available under the terms of the Creative Commons Attribution 4.0 International license (CC-BY 4.0).
