# Supplementary material for: Use of non-intrusive laser exfoliation to improve substance uptake into citrus leaves
Source: F1000Res. 2023 May 10;12:303. Originally published 2023 Mar 20. [Version 3] doi: 10.12688/f1000research.129789.3 (PMC10163338; doi:10.12688/f1000research.129789.3)
Supplement: Peer review history

# Peer review: Reviewer response for version 3

**DOI:** 10.5256/f1000research.147905.r172725

**Published:** 2023-05-11

**Authors:** Gabriel Bilmes

**Affiliations:** 1 Laser Ablation, Photophysics and 3D imaging Laboratory, Centro de Investigaciones Opticas (CONICETCIC-UNLP), La Plata, Buenos Aires, Argentina

No further comments to make.

Is the work clearly and accurately presented and does it cite the current literature?

Yes

If applicable, is the statistical analysis and its interpretation appropriate?

Not applicable

Are all the source data underlying the results available to ensure full reproducibility?

Yes

Is the study design appropriate and is the work technically sound?

Yes

Are the conclusions drawn adequately supported by the results?

Yes

Are sufficient details of methods and analysis provided to allow replication by others?

Yes

Reviewer Expertise:

Laser; laser spectroscopy; LIBS, Photoacoustics; Photophysics of biological systems; Laser Ablation, Laser cleaning, Lasers in conservation; 3D imaging;

I confirm that I have read this submission and believe that I have an appropriate level of expertise to confirm that it is of an acceptable scientific standard.

# Peer review: Reviewer response for version 3

**DOI:** 10.5256/f1000research.147905.r172724

**Published:** 2023-05-11

**Authors:** Humberto Cabrera

**Affiliations:** 1 Optics Lab, STI Unit, Abdus Salam International Centre for Theoretical Physics, Trieste, Italy

I don't have more comments, I recommend indexing.

Is the work clearly and accurately presented and does it cite the current literature?

Yes

If applicable, is the statistical analysis and its interpretation appropriate?

Partly

Are all the source data underlying the results available to ensure full reproducibility?

Yes

Is the study design appropriate and is the work technically sound?

Yes

Are the conclusions drawn adequately supported by the results?

Yes

Are sufficient details of methods and analysis provided to allow replication by others?

Yes

Reviewer Expertise:

Laser, optics, photothermal spectroscopy, digital holography microscopy

I confirm that I have read this submission and believe that I have an appropriate level of expertise to confirm that it is of an acceptable scientific standard.

# Peer review: Reviewer response for version 2

**DOI:** 10.5256/f1000research.146779.r167109

**Published:** 2023-05-05

**Authors:** Gabriel Bilmes

**Affiliations:** 1 Laser Ablation, Photophysics and 3D imaging Laboratory, Centro de Investigaciones Opticas (CONICETCIC-UNLP), La Plata, Buenos Aires, Argentina

This paper describes a laser-based “wax exfoliation” method to facilitate the penetration of substances, as agrochemicals, into plants leaves. The authors applied the method to citrus leaves showing its effectiveness, the non-invasive properties of laser exfoliation and the advantages over other methods. The proposed method is original and novel. The methodology followed to test it and the experiments carried out are adequate, and the results obtained are conclusive. The conclusions are well founded. For all these reasons, I consider that the work should be indexed only with minor formal modifications, which are detailed below.

- ROW 6 of the introduction “…located mainly on the less-exposed abaxial side of citrus leaves”. Consider modifying by: 
*located mainly on the less-exposed abaxial side of **plant** leaves.*
- Last paragraph of the introduction: “…through the application of a single laser beam”. Consider modifying by: 
*through the application of a single laser **shot.***
- In figure 1, both in the explanation and in the text, clarify how these images were taken, so that the treated part can be seen in yellow
- Row 4 after table 1: “...The infrared wavelength inherent to the erbium laser, which is identical to the absorption peak of water, allows for considerable laser-energy absorption by such water content.” Consider modifying by: 
*The infrared emission wavelength of 2940 nm from the erbium laser coincides with the maximum of the absorption peak of water. Therefore, given the high-water content of the leaf, the absorption of the laser energy is very high.*
- Consider reviewing the explanation of figure 3 in the text and the corresponding caption. It is not clearly written. Suggested wording: 
*Fluorescent microscopy images of the laser-based wax cuticle exfoliation from a citrus leaf: (a) the treated area of the abaxial of the leaf (upper side) is in green. The yellow -green points at the bottom of the figure correspond to the underlying epidermal walls autofluorescence;* *(b) Cross-section of a leaf* *’* *s laser treated area. Fluoresce of the Vascular bundles are visible.*
- Consider reviewing the caption of figure 5, it is confusing. Was the laser treatment applied to the entire leaf or only to the region where the spot appears? The caption seems to suggest that only the small marked region was treated. Clarify.

Is the work clearly and accurately presented and does it cite the current literature?

Yes

If applicable, is the statistical analysis and its interpretation appropriate?

Not applicable

Are all the source data underlying the results available to ensure full reproducibility?

Yes

Is the study design appropriate and is the work technically sound?

Yes

Are the conclusions drawn adequately supported by the results?

Yes

Are sufficient details of methods and analysis provided to allow replication by others?

Yes

Reviewer Expertise:

Laser; laser spectroscopy; LIBS, Photoacoustics; Photophysics of biological systems; Laser Ablation, Laser cleaning, Lasers in conservation; 3D imaging;

I confirm that I have read this submission and believe that I have an appropriate level of expertise to confirm that it is of an acceptable scientific standard.

# Response

**Published:** 2023-05-06

**Authors:** luis ponce cabrera

**Affiliations:** Onteko LLC, USA

The responses to the reviewer are included in bold letters following each question.

1. In figure 1, both in the explanation and in the text, clarify how these images were taken, so that the treated part can be seen in yellow.

**In the first paragraph of the results, an explanation about the color change observed in the irradiated area was included.**

1. Row 4 after table 1: “...The infrared wavelength inherent to the erbium laser, which is identical to the absorption peak of water, allows for considerable laser-energy absorption by such water content.”

Consider modifying by:

 The infrared emission wavelength of 2940 nm from the erbium laser coincides with the maximum of the absorption peak of water. Therefore, given the high-water content of the leaf, the absorption of the laser energy is very high.

**The paragraph was modified according to the reviewer's suggestion.**

1. Consider reviewing the explanation of figure 3 in the text and the corresponding caption. It is not clearly written. Suggested wording:

Fluorescent microscopy images of the laser-based wax cuticle exfoliation from a citrus leaf: (a) the treated area of the abaxial of the leaf (upper side) is in green. The yellow -green points at the bottom of the figure correspond to the underlying epidermal walls autofluorescence; (b) Cross-section of a leaf’s laser treated area. Fluoresce of the Vascular bundles are visible.

**The paragraph was modified according to the reviewer's suggestion.**

1. Consider reviewing the caption of figure 5, it is confusing. Was the laser treatment applied to the entire leaf or only to the region where the spot appears? The caption seems to suggest that only the small marked region was treated. Clarify.

**The caption of Figure 5 was modified to improve clarity.**

# Peer review: Reviewer response for version 2

**DOI:** 10.5256/f1000research.146779.r169728

**Published:** 2023-04-17

**Authors:** Humberto Cabrera

**Affiliations:** 1 Optics Lab, STI Unit, Abdus Salam International Centre for Theoretical Physics, Trieste, Italy

Authors clearly explained that the goal of the work  was to demonstrated the capabilities of the developed device and the specific application for laser exfoliation. Therefore, I agree that this goal was reached in a very elegant way. They also corrected properly the manuscript. I don't have any doubt that they will go ahead further with this development and will be a successful application. I would like to recommend indexing of the present work.

Is the work clearly and accurately presented and does it cite the current literature?

Yes

If applicable, is the statistical analysis and its interpretation appropriate?

Partly

Are all the source data underlying the results available to ensure full reproducibility?

Yes

Is the study design appropriate and is the work technically sound?

Yes

Are the conclusions drawn adequately supported by the results?

Yes

Are sufficient details of methods and analysis provided to allow replication by others?

Yes

Reviewer Expertise:

Laser, optics, photothermal spectroscopy, digital holography microscopy

I confirm that I have read this submission and believe that I have an appropriate level of expertise to confirm that it is of an acceptable scientific standard.

# Peer review: Reviewer response for version 1

**DOI:** 10.5256/f1000research.142501.r167110

**Published:** 2023-03-28

**Authors:** Humberto Cabrera

**Affiliations:** 1 Optics Lab, STI Unit, Abdus Salam International Centre for Theoretical Physics, Trieste, Italy

This manuscript describes a laser-based “wax exfoliation” method to facilitate the penetration of substances into the leaf and, together with enhancing their uptake into the phloem and subsequent transport across tissue. Authors demonstrated the effectiveness of the method to improve the penetration of foliar-applied substances into citrus leaves. One important property is to be non-invasive and more effective when compared to traditional laser treatment methods developed by same authors before using CO2 laser. The feasibility of the proposed method was validated by the experimental results obtained in citrus leaves. However, would be very interesting to see the results in the plant some period after treatment. The simple design without focusing lens and low cost laser makes this system attractive for practical applications. Some important discussions such as the productivity and practical massive implementation are not given. Discussions on other limitations of the system can better inform the readers on the potentials and applicability of this technology. Overall, this is a concise and clearly written manuscript and can be indexed after some major corrections listed below:

1. As a matter of comparison, I suggest to add a leaf without laser treatment (the same leaf prior to treatment) in Figure 1.
2. I would suggest statistical study about the plant grow *and *if it helps crop plants grow faster and helps to produce more crops, compared to non-treated plants *.*
3. How much affect the pulse duration on the efficiency of the exfoliation? Is it possible to do such study if the laser is available or there is no any sense? I suppose that with 200 μs the process will be mainly ablative, however some thermal effects can be also present. I suggest more detailed explanation in the text and description of the physical process.
4. In conclusion part authors stated that: *“This will include testing at longer distances based on the fact that the Er:YAG laser does not require a lens for this application.”* Considering the divergence of laser beam (mrad) I would like to know which will be such long distance (more than 30 cms) in order to keep the necessary energy densities and results achieved in this work.
5. Will be interesting if there is a description about efficiency and productivity compared with other methods. The laser beam is small, and if extensive zone are treated, the question about productivity and efficiency should be considered (when long distances and scanning procedure are applied).
6. Another important point here is also an estimation of the cost of the device or the prototype for massive implementation.

Is the work clearly and accurately presented and does it cite the current literature?

Yes

If applicable, is the statistical analysis and its interpretation appropriate?

Partly

Are all the source data underlying the results available to ensure full reproducibility?

Yes

Is the study design appropriate and is the work technically sound?

Yes

Are the conclusions drawn adequately supported by the results?

Yes

Are sufficient details of methods and analysis provided to allow replication by others?

Yes

Reviewer Expertise:

Laser, optics, photothermal spectroscopy, digital holography microscopy

I confirm that I have read this submission and believe that I have an appropriate level of expertise to confirm that it is of an acceptable scientific standard, however I have significant reservations, as outlined above.

# Response

**Published:** 2023-04-08

**Authors:** luis ponce cabrera

**Affiliations:** Onteko LLC, USA

General comments: The reviewer is correct that it would have been interesting to see the results in the plant for a period after treatment, but this is beyond the scope and objectives of this work. We are presenting a method that allows the uptake of substances to be created and have demonstrated that there is an improvement. However, the consequences of the treatments can be very different depending on the substance applied. Each substance will require a specific study as the doses and timing will be different.

 Regarding the productivity, it is also too early to tell as it will depend on how much substance is required and how many entry points will need to be opened in the plant. It may be sufficient to treat just one leaf in some cases, while in other cases it may be necessary to treat multiple leaves.

 Answers to the referee's questions:

1. Regarding the referee's suggestion to include a leaf before treatment in Figure 1, as can be seen in Figure 5, an image of the leaf where the treatment area is visible before (a) and after (b) treatment has already been included.
2. As mentioned in the general comments, a statistical study on how the plant grows and whether this helps farmers is beyond the scope of this article. This method can be used to improve the capture of many different substances, each of which will require a specific study.
3. Regarding the pulse duration, the available experimental setup does not allow for modification of that parameter, but we agree with the referee that it is worthwhile to conduct a study in the near future to optimize it. However, the penetration of the fluorescent glucose demonstrates that for the specific pulse duration we have used, the process significantly increases the capture of the substance, which was the main objective of this work.
4. We did not include a discussion of efficiency and productivity because the development of prototypes applicable under field conditions is future work. However, we have modified the final part of the conclusions to include an estimate that gives an idea of the feasibility of this method.
5. The answer to this question is related to the previous one. As mentioned earlier, the productivity for each specific substance will depend on many factors, and it is an analysis that is beyond the scope of this work.
6. The cost of the device will be related to parameters such as power, pulse energy, scanning system, and others, as well as the size of the market. A rough estimate could be a few thousand dollars. The financial viability will depend on how effective the method is in saving on agrochemicals or improving plant treatments. It is still too early to make an accurate economic evaluation.
